# Recent Advances in DNA Vaccines against Lung Cancer: A Mini Review

**DOI:** 10.3390/vaccines10101586

**Published:** 2022-09-21

**Authors:** Ting Huang, Li Liu, Zheng Lv, Kelei Zhao, Qiong Yi, Jing Zhang

**Affiliations:** 1 Antibiotics Research and Re-Evaluation Key Laboratory of Sichuan Province, School of Pharmacy, Chengdu University, Chengdu 610052, China; 2 Department of Oncology, Traditional Chinese Medicine Hospital of Meishan, Meishan 620010, China; 3 Department of Systems Biology, The University of Texas MD Anderson Cancer Center, Houston, TX 77030, USA

**Keywords:** DNA vaccines, lung cancer, tumor antigens, immunotherapy, adjuvants

## Abstract

Lung cancer is regarded as the major causes of patient death around the world. Although the novel tumor immunotherapy has made great progress in the past decades, such as utilizing immune checkpoint inhibitors or oncolytic viruses, the overall 5-year survival of patients with lung cancers is still low. Thus, development of effective vaccines to treat lung cancer is urgently required. In this regard, DNA vaccines are now considered as a promising immunotherapy strategy to activate the host immune system against lung cancer. DNA vaccines are able to induce both effective humoral and cellular immune responses, and they possess several potential advantages such as greater stability, higher safety, and being easier to manufacture compared to conventional vaccination. In the present review, we provide a global overview of the mechanism of cancer DNA vaccines and summarize the innovative neoantigens, delivery platforms, and adjuvants in lung cancer that have been investigated or approved. Importantly, we highlight the recent advance of clinical studies in the field of lung cancer DNA vaccine, focusing on their safety and efficacy, which might accelerate the personalized design of DNA vaccine against lung cancer.

## 1. Introduction

To date, lung cancer is one of the most diagnosed cancers and the leading cause of cancer death, which is a critical global issue [1]. In the last decade, the significant progress and transformed outcomes have been achieved in better understanding of cancer pathology, application of predictive biomarkers, and improved treatments for patients with lung cancer [2]. Despite the great recent efforts, over 1.7 million deaths and 2 million new cases per year for lung cancer are reported, even with the rapid increase in non-smokers [3]. Based on the histopathological features, lung cancer consists of two major subtypes, non-small-cell lung cancer (NSCLC) and small-cell lung cancer (SCLC), which account for approximately 80–85% and 15–20% of patients with lung cancer, respectively [4,5]. Cancer immunotherapies (CIs) are currently the most promising approach against cancer, which can contribute to overcome the drawbacks of conventional therapeutics [6]. CIs comprise CAR-T cell therapies, oncolytic viruses, antibody-based drugs, cancer vaccines etc. [7]. In this context, cancer vaccines represent an effective and promising scheme for manipulating the immune system.

Cancer vaccines are mainly divided into four types: tumor-cell-based vaccines, peptide or protein vaccines, viral-vector-based vaccines, and nucleic-acid-based vaccines (DNA or RNA vaccines) [8]. Among these potent vaccines, DNA vaccines are promising immune-therapeutics against cancers with various advantages. DNA vaccines are not only able to trigger innate immune responses, but also efficiently to induce both the humoral and cell-mediated immune responses of the host [9,10,11]. Additionally, a powerful DNA vaccine can consist of several genes encoding tumor-specific antigens, thereby augmenting immune responses against tumor antigens, which are involved in tumor initiation, progression, and metastasis [12]. For example, DNA vaccines have been extensively investigated for development of novel strategies against melanoma, prostate cancer, breast cancer, and lung cancer [11,12]. Moreover, DNA vaccines are easier to manufacture and have greater stability and safety compared to the traditional vaccines [13,14]. Although enormous efforts have been made to develop cancer vaccines, clinical therapies of cancer vaccines are still less efficacious because of the highly variable antigens of tumors and low immune responses [15].

In the current review, we briefly summarize important recent advances in the design of DNA vaccines targeting antigens of lung cancer and their significance in lung cancer treatment. Additionally, we discuss the novel strategies for improving antigen presentation and low immunogenicity, including new delivery platforms, molecular adjuvants, or immunomodulatory factors, and highlight the current clinical applications of DNA vaccines for lung cancer therapy. Finally, this article offers perspectives on the potential of DNA vaccines to overcome the major obstacles for lung cancer immunotherapies.

## 2. DNA Vaccines of Lung Cancer: Mechanisms of Immune Activation

**Figure 1:**
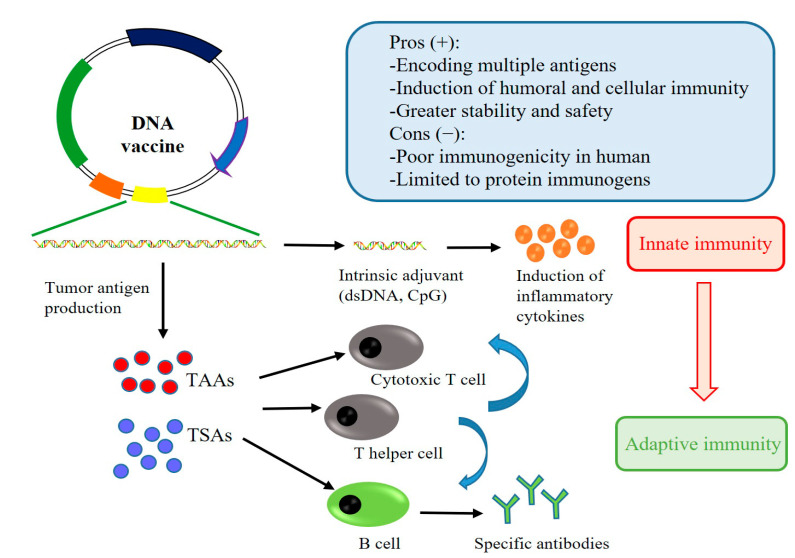
A scheme for the mechanisms of immune activation by DNA vaccines.

The principal concept of a DNA vaccine for lung cancer is to introduce potential and effective tumor antigens into the host and subsequently activate host immune responses to clear tumor cells (Figure 1). To create a powerful DNA vaccine for lung cancer, the specific tumor-antigen-encoding genes or encoded immunostimulatory molecules are cloned into a eukaryotic expression plasmid [16]. These vaccines can be delivered to the host using various vaccination routes, including intramuscular, intradermal, transcutaneous, and mucosal injections [17]. In addition, the DNA plasmids can be transported into the cells by physical methods including electroporation, sonoporation, or gene gun [18,19,20]. After taking up the plasmid, the host cell expresses the target antigen and presents the antigen to lymphocytes by the major histocompatibility complex (MHC) signaling pathways [21]. Afterwards, the exogenous antigens are presented to MHC class II molecules and inform CD4+ T cells to induce tumor-antigen-specific antibodies [22]. Likewise, the captured exogenous antigens can also be transferred to MHC class I molecules and induce specific cellular immune responses by interacting with CD8+ cytotoxic T cells, which is important for the clearance of tumor cells [14].

An effective immunity induced by the specific CD8+ T cell has an essential role in the antitumor activity, and DNA vaccines are powerful at inducing CD8+ T cell responses [23]. Just two cancer vaccines are currently approved for human application (Sipuleucel T and T-VEC). Sipuleucel-T (Provenge) was the first dendritic-cell-based cancer vaccine approved by the U.S. Food and Drug Administration (FDA) for the control of prostate cancer, and T-VEC (talimogene laherparepvec) was the first oncolytic virus vaccine for the treatment of patients with melanoma [24,25,26]. Notably, an interesting preclinical study demonstrated that a genetically engineered DNA vaccine induced highly antitumor efficacy and decreased the tumor nodules in a mouse lung cancer model [27]. Although DNA vaccines are shown to elicit therapeutic antitumor immune responses, the DNA vaccines for lung cancer are still in the clinical trial phases. In this context, new developments in DNA plasmid delivery and optimization will improve the efficacy of DNA vaccines in clinical studies to achieve the goals of translational applications in patients.

## 3. Potent Antigens Selection for Lung Cancer

The most vital process in the development of DNA vaccine for lung cancer is to select the potent tumor antigens. These antigens are homogenously expressed in the cancer cells and are designed to prevent tumor immune escape and cancer metastasis. Additionally, the antigens are highly immunogenic and are indispensable for cancer cell survival [28]. To the best of our knowledge, tumor antigens are divided into two major categories, including tumor-associated antigens (TAAs) and tumor-specific antigens (TSAs) [29]. For example, many TAAs are monomorphic self-antigens, which are expressed in both cancer cells and normal host cells. TSAs (neoantigens) are encoded by tumor-specific somatic mutations and are particularly expressed in neoplastic cells [30].

In the past few years, it was extensively investigated that TAAs showed less clinical benefit because of the preexisting central tolerance to self-antigens [28]. Especially, targeting TAAs (MAGE-A3, MUC-1) in lung cancer induced inefficient immune response in the phase III trials [31]. In contrast to TAAs, TSAs are less likely affected by central or peripheral tolerance due to molecular alterations [32,33]. TSAs are easily generated because of the genomic instability presented in lung cancer, and are effectively identified by immune system of the host [30]. Thus, the generation of TSAs will contribute to designing tumor vaccines, such as identification of TSAs by rapid genomic profiling and computational prediction pipelines based on next-generation sequencing [33]. However, not all the genomic instability exhibits similar effects such as new neoantigen generation and host response to cancer immunotherapy [34]. Tumor mutation burden (TMB) is considered as a promising biomarker of response to immune checkpoint inhibitors, while it is hard to detect neoantigen load in clinical practice due to biological or economic problems [35].

With the development of next-generation sequencing and relevant cutting-edge bioinformatics, more and more unique TAAs and TSAs have been identified for lung cancer vaccines [36,37,38]. Given that both TAAs and TSAs are of considerable importance in T-cell-mediated antitumor immunity, targeting these potential antigens is critical for cancer immunotherapy. Herein, we summarize the updated TSAs and TAAs associated with lung cancer in Table 1 and Table 2. Since most antigens of lung cancer come from self-tissue, innovative engineered DNA vaccines with novel antigens will surmount inherent immune tolerance and inefficient immune response as mentioned above.

## 4. DNA Vaccine Delivery Platforms

A major challenge in the development of DNA vaccines is to ensure the delivery of DNA plasmids to the appropriate cells and tissues. Thus, choosing an applicable delivery system is thought to be a key factor to induce the activation of host immune system and to reduce some side effects [11]. In this section, we summarize the recent advances in DNA vaccine delivery platforms for cancer including electroporation and gene-gun-based delivery, nanoparticle-based delivery, and self-assembling peptides-based delivery systems.

Electroporation is one of the most intensively studied strategies to promote the delivery of DNA plasmids into antigen-presenting cells (APCs) [61]. In terms of mechanism, electroporation delivery can increase cell permeability by forming transient pores, thus allowing more DNA plasmids to enter into the cells [62]. In addition, electroporation has an adjuvant effect to attract specific immune cells such as dendritic cells to the DNA injection sites, and subsequently triggering the production of proinflammatory cytokine and the magnitude of cancer antigen-specific immune response [63]. A number of clinical trials have tested the delivery efficacy of the DNA vaccine against cancers using electroporation such as melanoma, prostate cancer, and colon adenocarcinoma [12,64]. Another common scheme is applying a gene gun to transfer DNA vaccine, which is coated with a gold particle. In this delivery system, the cytotoxic T lymphocyte responses can be enhanced and less DNA is required in different experimental settings [65]. Numerous convincing evidence have demonstrated that the antitumor effects of cancer vaccines can be enhanced by gene gun delivery against various cancers such as lung cancer [66,67,68,69,70,71]. In spite of this significance, electroporation or gene-gun-based delivery suffer some drawbacks, such as causing considerable pain on administration and not being suitable for community-wide vaccination [63].

An alternative delivery approach has been designed to enhance the uptake of target DNA vaccine using nanoparticle-based drug delivery platforms. This novel delivery platform can overcome the pharmacokinetic limitations and improve the poor bioavailability or solubility of drugs. Up to now, various nanoparticles have been applied to deliver DNA-based vaccines and promote antitumor immune responses, including polymeric nanoparticles, liposomes, silica nanoparticles, bisphosphonate-modified calcium phosphate nanoparticles, gold nanoparticles, virus nanoparticles, and carbon nanotubes [72,73,74]. Liposomes are one of the well-recognized nanoparticle vaccine delivery platforms, which are easy to construct by altering the composition, surface of lipid, and other important properties [75,76,77]. Liposomes can not only augment the immunogenicity of special antigens for cancer DNA vaccines, but they also improve the therapeutic efficacy. BioNTech have developed a novel nanoparticle for gene delivery called Lipoplex, which is mainly complexed with RNA encoding target tumor antigens for cancer immunotherapy [78]. In addition, Lipoplex complexing with DNA plasmid reduced tumor proliferation and tumor angiogenesis of lung cancer [79].

Self-assembling peptides (SAPs) are small biomedical materials that can also function as an effective drug delivery system to deliver antigens to cancer cells [80,81]. SAPs can be manufactured to form various constructions such as nanomicelles, nanotubes, nanovesicles, nanotapes, and hydrogels [80]. This innovative delivery system has several advantages compared to liposomes or nanoparticles, such as high drug-loading efficiency, low drug leakage, easier uptake, and highly biodegradable properties [63]. Additionally, it can induce long-time immune response in an adjuvant independent manner [81]. Recently, an interesting study showed that a new delivery platform based on SAPs, called the Glycosaminoglycan (GAG)-binding enhanced transduction (GET), was able to promote delivery of nucleic acids for gene therapy in lung organs [82]. The tripeptide complexes the DNA plasmids into the novel nanoparticles and can be delivered to various organs, in particular in the lung, and has great potential application in delivery of DNA vaccines. Nevertheless, the main drawback associated with low pH in SAPs is that it needs to improve [83]. For instance, the SAPs might damage cells or host tissues at low pH condition, and keeping constant neutral pH can contribute to embedding molecules in SAPs during the self-assembly process. Moreover, the temperature condition alters the self-assembly behavior when designing the novel SAPs, and also needs to be optimized [80].

## 5. Adjuvants

DNA vaccines of lung cancer induce a systemic immune response, including humoral and cellular immune responses, and are effective to prevent the metastases of tumors. Additionally, DNA vaccines activate immunological memory of host immune cells compared to small molecule inhibitors and antibodies [11]. Nevertheless, single antigen in a DNA vaccine might induce poor adaptive immune response [9]. Therefore, it is necessary to develop new DNA vaccine adjuvants, which can target important elements of the immune system to elicit a robust and sustained immune response [63]. Here, we summarize the recent advances in new DNA vaccine adjuvants for lung cancer, including inherent adjuvants and molecular adjuvants [63,84,85,86,87,88].

The inherent adjuvants contain pathogen-associated molecular patterns (PAMPs) such as CpG motifs and lipopolysaccharides, and dendritic cells. The PAMPs are normally recognized by the Toll-like receptors (TLRs) [63]. TLR agonists are considered as a potential vaccine adjuvant, which can mimic the progress of microbial infection and increase the immune efficacy of a cancer vaccine [48,89,90]. To date, several TLR agonists are in clinical trials as promising vaccine adjuvants for cancer therapy, including TLR3 agonist, TLR4 agonist, TLR7 agonist, TLR8 agonist, and TLR9 agonist [48,89,90,91]. Among these TLR agonists, two TLR agonists were approved as an innovative vaccine adjuvant, including Monophosphoryl lipid A (MPL) and CpG oligodeoxynucleotides (ODN) 1018 [48]. MPL is a detoxified LPS derivative that can activate the TLR4 signaling pathway and is applied as an important element of the Cervarix vaccine of the year 2009 [92]. The ODN 1018 is a TLR9 agonist and is used for an adjuvant of the Heplisav-B vaccine that followed in 2017 [93].

There is emerging interest in the use of molecular adjuvants such as cytokines and chemokines to enhance the efficacy of a cancer vaccine [63]. These adjuvants can attract specific immune cells to the location of injection or mediate the traffic of antigen such as RANTES, CCL5, and CXCR2 [94,95]. Moreover, they can enhance the immunogenicity of target antigens in host cells and modify the immune responses against various pathogens such as IFN-γ, IL-2, IL-12, IL-15, and granulocyte-macrophage colony stimulating factor (GM-CSF) [96,97,98]. GM-CSF is one of the well-recognized immunostimulatory factors and has been investigated in many clinical trials of cancer vaccines [98,99]. A recent study found that co-delivery of dendritic cells and the modified oncolytic adenovirus expressing IL-12 and GM-CSF cytokines induced a strong antitumor immune response [100]. Another class of potential vaccine adjuvants is the CD40 agonists and stimulator of interferon genes protein (STING) agonists [101,102,103,104]. CD40 is normally expressed on antigen-presenting cells (APC) or macrophages, and CD40 agonists (CD40a) can activate maturation of APCs for enhancing tumor-specific antigen presentation [104]. A number of trials have shown that CD40 agonists were applied in combination with CSF1R inhibitor or TLR agonists for cancer therapy in a vaccine-adjuvant way [101,103,104]. STING is an important transmembrane protein that is located in the endoplasmic reticulum and in response to cytosolic DNA [105]. STING agonists exhibited effective antitumor activity, including cyclic di-guanosine monophosphate and synthetic cyclic dinucleotide derivatives [106]. A number of studies demonstrated that STING agonists combined with a cancer vaccine or delivery platform induced a potent inflammatory response and modulated the tumor microenvironment by increasing proliferation of CD4+ T cell [107,108,109,110]. Although STING agonists might cause some systemic toxicity, the novel strategies of combined therapy would accelerate their application as a cancer vaccine adjuvant.

## 6. Recent Clinical Trials Based on DNA Vaccines against Lung Cancer

DNA vaccines elicited great immune responses in various animal models against cancers [12]. For instance, more than five different DNA vaccines are licensed and employed in the veterinary industry, and one of the DNA vaccines (Oncept vaccine) in particular is applied to treat canine melanoma based on a xenogenic antigen [21,111,112]. Many clinical trials have investigated the efficacy and safety of designed DNA vaccines against a majority of tumor models, including breast cancer, cervical cancer, pancreatic cancer, prostate cancer, and lung cancer [16]. However, none of these DNA vaccines have been approved for clinical application in lung cancer patients by the FDA or relevant agencies around the world. A powerful search for the studies of clinical trials with “lung cancer” and “DNA vaccines” in a public database (www.clinicaltrials.gov (accessed on 8 August 2022)) [113] showed that approximately 10 studies were detected in the last decade under the following conditions: “completed”, “withdraw”, “recruiting”, “not yet recruiting”, and “terminated”. Among these clinical trials employing DNA vaccines against lung cancer, only several trials have published the results. In this section, we summarize the recent advances in clinical studies of DNA vaccines for the treatment of lung cancer.

The NCT02179515 phase I clinical study investigated the safety and effectiveness of MVA-brachyury-TRICOM vaccine, which was based on a Modified Vaccinia Ankara (MVA) vector and expressing three important human costimulatory molecules (B7.1, ICAM-1, and LFA-3, abbreviated as TRICOM) for treatment of lung cancer and other tumors [114,115]. A total of 38 cancer patients were injected subcutaneously with different doses of MVA-brachyury-TRICOM at monthly (approximately 28 days) intervals for 3 months. This novel vaccine effectively activated specific CD8+ and CD4+ T cells in human dendritic cells in vitro assay. Notably, no obvious dose-limiting toxicities were detected in patients, and brachyury-specific T cell responses were highly induced at different dose levels and in many patients. A recent study reported the results of a phase I study (NCT00199849), in which 4–8 µg dosages of a pPJV7611 plasmid coding for the NY-ESO-1 protein were delivered by a particle-mediated epidermal delivery (PMED) method in patients with NSCLC. A general increase in antibody titer and NY-ESO-1-specific CD4+ and CD8+ T cells were detected in most patients. In addition, a positive delayed-type hypersensitivity (DTH) reaction to NY-ESO-1 protein was not observed in all patients [116,117]. In another phase I study (NCT00423254), the safety and immune responses of pPRA-PSM DNA vaccine combined with synthetic peptides E-PRA and E-PSM were evaluated for the treatment of solid malignancies such as small-cell lung carcinoma. The treatment was well tolerated and safe. More than half of the patients exhibited the intensification of immune response by detecting the proliferation of PRAME-specific or PSMA-specific T cells isolated from the blood. Importantly, some patients exhibited stable disease for 6 months or even longer [116,118]. Except for the completed clinical trials, there are about five ongoing clinical trials using DNA vaccines against lung cancer [119]. They are all in clinical phase I or phase II and use DNA vaccines as naked plasmid or combined with drugs such as durvalumab for the treatment of lung cancer. Nevertheless, the results for these trials are not available to date.

Interestingly, eight studies were found in PubMed (pubmed.ncbi.nlm.nih.gov (accessed on 9 August 2022)) following these criteria: “lung cancer”, “DNA vaccine”, article type “clinical trial”, from 2001 to 2022 [120]. Among these clinical trials, most of them concentrated on prophylactic DNA vaccine or molecular adjuvant against cancer [121,122]. For example, CpG ODN (K3), a novel synthetic DNA adjuvant, was used to boost the immune response in patients with lung cancer. CpG ODN (K3) activated innate immunity by increasing the secretion of IFN-γ, CXCL10. Additionally, Th1-type immune response and cytotoxic activity were significantly enhanced in patients. Taken together, DNA vaccines exhibited great potential against lung cancer compared to conventional therapies. Among the published clinical trials, few obvious side effects were observed, and a significant increase of specific antibody or CD8+ T cells was detected by the novel DNA vaccines. Although many DNA vaccines encoding novel TAAs or TSAs were presented in the preclinical or clinical studies, only a few personalized antigens were detected. More clinical trials are required to further explore their mechanism of action and clinical practice.

## 7. Future Prospects

DNA vaccines are the next generation of immunotherapy that consists of immunoenhancement and precision medicine. In the context of DNA vaccines, they can provide therapeutic strategy for a majority of cancers, including lung cancer. However, despite the remarkable progress of DNA vaccines in cancer research, DNA vaccines reveal some limitations and challenges in trials, including poor immunogenicity in humans, limited to protein immunogens, and inducing passible antibody production against DNA. In addition, the early designs of DNA vaccines and immunologic tolerance are the main causes for failure of DNA vaccines in human clinical trials.

Recent breakthroughs have been investigated to enhance the immune response of host against lung cancer by adding the TAAs or TSAs and adding the novel immunological adjuvants such as cytokines and chemokines. Although intramuscular injection of DNA vaccines is a common route, device-mediated immunotherapy is also general, especially electroporation and gene gun. In addition, nanoparticle-based delivery and molecular-adjuvant-based DNA vaccine showed increased potential efficacy in numerous ongoing clinical trials. The recent clinical trials imply that the current cancer vaccines are not sufficient to provide excellent outcomes only by a single component. Therefore, combinations with other strategies, such as adding novel adjuvants and delivery platform, would improve the clinical outcomes compared with the monotherapy. Furthermore, the personalized tactic in the DNA vaccine design will be critical for success in the clinical applications. To accomplish the applicable treatment for the patients with lung cancer by DNA vaccine immunization, more detailed studies are needed in the future.

## Figures and Tables

**Table 1:** Potential neoantigens identified in lung cancer.

No. of Potential NEOANTIGENS	Patient	Parameter (s)	Identified Year	References
8–610	34	MHC-I binding affinity	2015	[39]
288–417	2	MHC-I binding affinity	2016	[40]
80–741	7	MHC-I binding affinity	2016	[41]
102–316	4	MHC-I binding affinitySelf-similarity	2017	[42]
12	147	MHC-II binding affinity	2018	[43]
1–219	12	MHC-I binding affinityAntigen processing	2018	[44]
1–139	14	MHC-I binding affinity	2019	[45]
54–2992	20	MHC-I binding affinity	2020	[46]
12–30	12	MHC-I/MHC-II binding affinity	2021	[47]
225	24	MHC-I/MHC-II binding affinity	2021	[48]

Abbreviation: MHC, major histocompatibility complex.

**Table 2:** Potential TAAs identified in lung cancer.

TAAs	Gene Function	Histology	Identified Year	References
OLFM1	Suppress cell growth and metastasis	LC	2015	[49]
SQLE	Cell proliferation and metastasis	LC	2015	[49]
c-Myc	Cell growth and metabolism	LC	2016	[50]
HNRNPA2B1	mRNA metabolism and transport	LC	2017	[51]
ENO1	Regulate cell proliferation and metastasis	NSCLC	2017	[52]
P53	Inducing cell cycle arrest, and DNA repair	LC	2017	[53]
GBU4-5	Cell growth and division	SCLC	2018	[54]
IGFBP-1	Cell migration	LC	2019	[38]
FGFR1	stem cell leukemia/lymphoma syndrome	LC	2019	[55]
CA125	Cell adhesion, migration, and invasion	NSCLC	2019	[56]
GAGE7	Influence cancer progression	NSCLC	2019	[57]
TOP2A	Metabolism for proteins and DNA damage	LC	2020	[58]
SOX2	Cell proliferation, metastasis, and drug resistance	LC	2020	[59]
CAGE	Cell cycle, growth, and proliferation	NSCLC	2021	[60]

Abbreviations: TAAs, tumor-associated antigens; LC, lung cancer; NSCLC, non-small-cell lung cancer; SCLC, small-cell lung cancer.

## Data Availability

Not applicable.
